# Knowledge, attitude and practice of breast self-examination among female undergraduate students in the University of Buea

**DOI:** 10.1186/s13104-015-1004-4

**Published:** 2015-02-15

**Authors:** Fon Peter Nde, Jules Clement Nguedia Assob, Tebit Emmanuel Kwenti, Anna Longdoh Njunda, Taddi Raissa Guidona Tainenbe

**Affiliations:** Department of Nursing, Faculty of Health Sciences, University of Buea, P.B. 63, Buea, Cameroon; Department of Biomedical Sciences, Faculty of Health Sciences, University of Buea, P.B. 63 Buea, Cameroon; Department of Medical Laboratory Sciences, Faculty of Health Sciences, University of Buea, P.B. 63 Buea, Cameroon; Department of Public Health and Hygiene, Faculty of Health Sciences, University of Buea, P.B. 63 Buea, Cameroon

**Keywords:** Breast cancer, Breast self-examination, Knowledge, Attitude, Practice, Undergraduate students, University of Buea, Cameroon

## Abstract

**Background:**

The incidence of breast cancer is on the rise in many parts of Africa. In Cameroon, there were an estimated 2625 cases per 100,000 in 2012. The awareness of breast cancer preventive methods is therefore critical in the reduction of breast cancer morbidity and mortality. This study evaluated the knowledge, attitude and practice of breast self-examination (BSE), among female undergraduate students in the University of Buea.

**Methods:**

The study comprised 166 female students of ages 17-30years (mean = 22.8 ± 3) sampled randomly. Data was collected by a pretested self-administered questionnaire.

**Results:**

Nearly three quarter (73.5%) of the respondents had previously heard of BSE. Only 9.0% knew how to perform BSE. Similarly, only 13.9% knew what to look for while performing BSE. Television (19.9%) was the main source of information on BSE. Although perceived by 88% of the respondents as important, only 3% had performed BSE regularly. Furthermore, only 19.9% of the respondents have been to any health facility to have breast examination. Overall, although a majority (63.3%) of the respondents had a moderate attitude towards BSE as an important method for early detection of breast cancer, just a modest 9.6% were substantially aware of it. Lack of knowledge on BSE was cited as the main reason for not performing BSE. A significant association was observed between knowledge and the practice of BSE (P = 0.029), and between attitude and the practice of BSE (P = 0.015).

**Conclusions:**

These findings highlight the current knowledge gap that exists in the practice of BSE in the prevention of breast cancer in the study population. Sensitization campaigns and educational programmes ought to be intensified in order to address this issue.

**Electronic supplementary material:**

The online version of this article (doi:10.1186/s13104-015-1004-4) contains supplementary material, which is available to authorized users.

## Background

Breast cancer is the most common cancer among women in developed and developing countries [1]. Worldwide, over 1.15million cases of breast cancer are diagnosed every year [1], and 502,000 women die from the disease each year, making it second only to lung cancer as the cause of cancer related deaths among women [2,3]. Breast cancer incidence has been on the rise in many parts of Africa – studies performed in Cameroon [3] and Ghana [4] revealed that breast cancer is the most common malignant cancer in women. In Cameroon, the incidence of breast cancer was estimated at 2625 per 100,000 in 2012 [5]. Breast cancer is becoming an increasing urgent problem in low-resource regions where incidence rates have been shown to increase each year by as much as 5% [6].

The risk factors for breast cancer include advancing age [7], women with history or family history of breast cancer [8], women who started menstruation early or went through menopause late [9,10], and the use of hormonal replacement therapy (HRT) with combined estrogen and progesterone [11,12]. Breast cancer in the early stages typically do not produce symptoms but as the tumour enlarges, symptoms produced include; painless lump in the breast, lump under the armpit, breast pain, swelling or thickness of the breast’s skin, spontaneous discharge of the nipple particularly if bloody and erosion or inversion in the nipple [7].

The control of breast cancer in most developing countries including Cameroon is under the auspices of national control programmes promoted by the WHO [1], and this involves educating and screening young women for signs of breast cancer. The earlier breast cancer is detected, the better the effectiveness of the treatment and the likelihood of survival [1]. Breast cancer screening methods include breast self-examination (BSE), clinical breast examination and mammography, and these are usually done in combination [13]. Among these methods, mammography is the only method that has been proven to be effective, but the method is very costly, and is cost-effective and feasible in countries with good health infrastructure [1,7]. BSE is the recommended method in developing countries because it is easy, convenient, private, safe and requires no specific equipment [14,15]. Its purpose is to make women familiar with both the appearance and feel of their breasts as early as possible, so that they will be able to easily detect changes in their breast. Several studies have revealed that a positive association exists between the performance of BSE and detection of breast cancer [16], and most of the early breast tumour detection have been self-discovered [17]. In Cameroon, cancer surveillance system is not well organized as many deaths related to cancer are not reported nor recorded. Control of cancer including breast cancer is through the organisation of periodic screening campaigns which are often not very effective since they are organized in only the major urban centers [18]. Buea like most semi-urban areas in Cameroon, women often learn of BSE from health personnel. Sensitization through the media including radio, television are rare.

BSE for the early detection of breast cancer is not often done by women. In studies performed by Godazandeh [19], and Nafissi et al. [20], only 17% and 12% of women respectively were observed to perform BSE monthly. This does not differ from what transpire among health personnel. In one study, only 14% of nurses and midwifes were observed to regularly perform BSE [21]. As a result, most cases of women diagnosed with breast cancer are usually in an advanced stage of the disease. In a study performed in Ghana, 70% of women who were diagnosed with breast cancer were already in an advanced stage [22]. Although BSE is a simple, quick and cost-free procedure, it appears that many women either perform it incorrectly or not at all. The purpose of this study was to evaluate the knowledge, attitude and practice of BSE among female undergraduate students of the University of Buea, in order to generate data that may be useful in designing interventions aimed at creating awareness of BSE as a screening method for the early detection of breast cancer.

## Methods

### Study design and settings

In a cross sectional descriptive study which lasted from April to July 2014, female undergraduate students were randomly selected in the University of Buea (commonly referred to as UB). UB is located in Buea (coordinates: 4°10′N 9°14′E) in the South West region of Cameroon. The University of Buea is one of the two Anglo-Saxon state universities in Cameroon and is a center of attraction for youths who either move there for studies or benefit from the economy triggered by the presence of the university.

### Data collection

Participants were selected proportionately according to the population of the various academic levels by simple random sampling. Written informed consent were obtained from all the participants prior to data collection.

Data were collected using a self-administered semi-structured questionnaire. The questionnaire was pretested using ten (10) students selected from a neighboring higher institution of learning before the final study. Printed copies of the questionnaire were handed to the respondents in person. To ensure confidentiality, no name was collected, instead codes were used to identify the respondents. Furthermore, the respondents were also provided with an envelope to seal the questionnaire upon completion before submission.

Ethical approval for the study was obtained from the Faculty of Health Sciences Institutional Review Board (FHS IRB) of the University of Buea, Cameroon. Administrative clearance was obtained from the Delegation of Public Health, South West Region of Cameroon.

### Assessment of knowledge on BSE

There were 14 knowledge indicators used to evaluate the respondents. Knowledge was scored on 14, one for each indicator. Respondents who scored between 10 and 14 were considered as substantially aware of BSE, scores between 7 and 9, as partially aware, and scores between 0 and 6 as not aware.

### Assessment of attitude towards BSE

There were 10 attitude indicators used to evaluate the respondents. Attitude was scored on 20, 2 for every response to the indicators which demonstrated that the respondent was highly in favour of BSE and 1 for every response which demonstrated that the respondent was partially in favour of BSE and 0 for response that was not in favour of BSE.

### Data analysis

Data from the questionnaire was processed using Epi Info version 7.1.3.0 (Epi Info™, CDC) and analysed using statistical package for the social sciences (SPSS) version 17.0 (SPSS Inc., USA). Statistical analysis performed included the Pearson Chi-square test to determine the association between knowledge and attitude stratified according to the scores, and the practice of BSE. Statistical significance was set at P < 0.05.

## Results

### Characteristic of study population

One hundred and eighty two (182) students were selected to participate in the study and 166 (91.2%) completed and returned the questionnaires. The respondents were between 17 and 30 years (mean ± SD = 22.8 ± 3) of age. Among the 166 respondents, 151 (91%) were still single while 15(9%) were married. Five (3%) of the 166 respondents had a family history of breast cancer.

- Knowledge on BSE

Nearly three-quarter (73.5%) of the respondents had heard about BSE before (Table 1). Approximately 4 in 10 (37.3%) of the respondents were aware that BSE should be performed monthly. Very few (9.0%) of the respondents actually knew how to perform BSE. Furthermore, only a few (13.9%) knew what to look for while performing BSE. A majority (88.6%) of the respondents perceived BSE as an important technique in the early detection of breast cancer. The other indicators are summarized in Additional file 1: Table S1. Overall, just a modest 9.6% of the respondents were substantially aware of BSE, 53% were partially aware, and 37.4% had never heard of BSE.

**Table 1:** **Knowledge and sources of information on BSE among the 166 respondents**

**Knowledge**	**Responds**	**Frequency**	**%**
Ever heard of BSE	Yes	122	73.5
	No	44	26.5
Know BSE should be performed monthly	Yes	62	37.3
	No	104	62.7
Know the 3 positions to perform BSE (lying down, standing in front of mirror, showering)	Yes	15	9.0
	No	151	91.0
Know what to look for while performing BSE	Yes	23	13.9
	No	143	86.1
Know BSE is important in the early detection of breast cancer	Yes	147	88.6
	No	19	11.4
Overall awareness of BSE*	Substantially aware	16	9.6
	Partially aware	88	53.0
	Not aware	62	37.4
Sources of information on BSE	Television	33	19.9
	Friends	32	19.3
	Physicians	29	17.5
	Family	22	13.3
	Teacher	18	10.8
	Nurse	16	9.6
	Radio	14	8.4
	Magazine	9	5.4
	Newspaper	6	3.6
	Internet	6	3.6
	Pamphlets	4	2.4

*Knowledge on BSE was scored on 14. Substantially aware was considered as score between 10 and 14; partially aware, 7–9; and not aware, 0–6.

The main sources of information on BSE cited by the respondents were television (19.9%), friends (19.3%) and doctors (17.5%) (Table 1).

b) Attitude towards BSE

Approximately 6 in 10 (59%) of the respondents were in accord that they can actually detect breast cancer by themselves (Table 2). Half (51.4%) admitted that they were not afraid to detect breast cancer meanwhile 26.5% were afraid. A majority (88%) of the respondents approved that BSE was important and useful in the early detection of breast cancer. A majority (81.9%) of the respondents cited that they were motivated by publicity and campaigns to perform BSE. Approximately 8 in 10 (77.1%) of the respondents did not consider BSE as a “disgraceful” practice. The other indicators used to evaluate the attitude of the respondents are summarized in Additional file 2: Table S2. Overall, 34.3% of the respondents were highly in favour of BSE, 63.3% moderately in favour, and only 2.4% were not in favour.

**Table 2:** **Attitude of the 166 respondents towards BSE**

**Attitude**	**Response**	**Frequency**	**%**
You can find breast cancer by yourself	Agree	98	59
	Disagree	55	33.1
	Unsure	13	7.8
You are afraid that you'll detect breast cancer	Agree	44	26.5
	Disagree	102	61.5
	Unsure	20	12
Screening for abnormality of BSE is important and useful	Agree	146	88
	Disagree	4	2.4
	Unsure	16	9.6
Publicity or campaigns motivate you to detect breast cancer by yourself	Agree	136	81.9
	Disagree	17	10.2
	Unsure	13	7.8
BSE is a "disgraceful" practice in that other people see or touch the breast to detect breast	Agree	23	13.9
	Disagree	128	77.1
	Unsure	15	9
Overall attitude towards BSE*	High	57	34.3
	Moderate	105	63.3
	Low	4	2.4
Factors that influences the respondents to perform BSE	Family history of breast cancer	3	1.8
	Ordered by health personnel	25	15.1
	Electronic or print media influence	19	11.4
	Peer	21	12.7
	Family	8	4.8

*Attitude was scored on 20. High attitude was considered as score between 17 and 20; Moderate attitude, 10–16; and Low attitude, 0–9.

Health personnel (15.1%) and peers (12.7%) were cited by the respondents as the main factors that influenced them to perform BSE (Table 2).

c) Practice of BSE

Only 62 (41%) of the 166 respondents in this study had ever performed BSE, 49 (29.5%) claimed to have performed BSE within the past 12 months. Only 5(3%) had performed BSE regularly (10-12 times) within the past 12 months.

Among the 49 respondents who had performed BSE within the past 12 months, 9 (18.4%) admitted to have noticed one or more of the following breast abnormalities: abnormal pains 2 (22.2%), abnormal lump 1 (11.1%), discharge of pus from the nipple 1 (11.1%), abnormal size increase 1 (11.1%) and others 4 (44.4%).

In this study, only 19.9% of the respondents had ever been to any health facility to have their breast examined. Only 36.1% of the respondents said that they had encouraged other people to practice BSE.

The main reason for not performing BSE as cited by the respondents were the lack of knowledge 73 (44%); followed by the reason that the respondents did not have any signs of breast cancer 61 (36.7%); forgetfulness 33 (19.9%); lack of time 16 (9.6%); fear of finding lumps 12.9 (7.8%); and embarrassment 8 (4.8%).

In this study, a significant association was observed between knowledge and the tendency to perform BSE (χ^2^ = 6.98, df = 4, P = 0.029), and between attitude and the tendency to practice BSE (χ^2^ = 10.58, df = 4, P = 0.015).

## Discussion

As mentioned earlier, the incidence and mortality due to breast cancer is on the rise in many parts of Africa. Breast cancer is preventable if detected early enough [1]. There are several methods by which the early onset of breast cancer can be detected including breast self-examination. Although there are some controversies regarding the techniques used in performing BSE, the method is still considered as relevant, and is therefore recommended in developing countries where access to diagnostic and curative facilities may be a problem [14,15]. The rising trend in the incidence of breast cancer in Africa may be an indication that many young women still do not screen for the early detection of breast anomaly. This study performed among female students in the University of Buea was aimed at evaluating their knowledge, attitude and practice of BSE.

The level of practice of BSE observed in this study was generally low. Only three in ten of the students had performed BSE within 12 months prior to the study. Moreover, only 2 in 10 of the students have ever been to any health facility to have their breast clinically examined. Similar findings have been reported among students in Malaysia [23]. Furthermore, among those who had performed BSE before, only 3% practiced it regularly on a monthly bases, which is not different from what was observed in the study in Malaysia [23]. An earlier study performed among women in Buea had also revealed that women did not perform BSE regularly [3]. The main factor that could be attributed to this is the lack of knowledge on BSE which was generally observed to be unsatisfactory, with only 9.6% of the respondents substantially aware of BSE as a method. Lack of knowledge has also been implicated as the main reason for the poor practice of BSE in similar studies performed elsewhere [24,25]. A majority (88.6%) of the respondents in this study perceived BSE as an important technique in the early detection of breast cancer, but just 9% knew how to perform it, and only 13.9% knew what to look for while performing BSE. Similar observations have been reported elsewhere [23,25]. The main source of information on BSE as cited by the respondents in this study was television which is not different from what has been observed in studies performed elsewhere [23,25-27]. This finding shows that the media especially television can be used to sensitize women on the importance of BSE, as well as instruct women on how to perform BSE.

Physicians, nurses and other health personnel also have a role to play in sensitizing and educating young women on the importance of BSE and how it should be performed, which is evident in this study as 15.1% of the respondents cited health personnel and peers as their main influence for practicing BSE. This observation is in accordance with the study among university students in Ghana [25]. The general attitude of the respondents in this study towards BSE was moderate, implying that just a little motivation may easily sway their attitude towards highly in favour of practicing BSE. Motivation to practice BSE could be through the organisation of health campaigns and publicity as was observed in this study. Fear of detecting breast cancer was one of the factors cited by the respondents for not practicing BSE. Educating these young women could also help instill some courage in them. Further studies will be required to throw more light on the role of health personnel and the media on the uptake and practice of BSE in women in the study area.

Although this study has revealed the inadequate knowledge on the practice of BSE by female undergraduate students in the University of Buea, as well as provided information on the possible methods that can be used to improve on the interest and practice of BSE among young women, the study is however limited in that it is confined to a sample of young educated women in a semi-urban area which does not necessarily reflect what transpire among women in rural areas. Furthermore a majority of students in the University of Buea are from the two English speaking regions of Cameroon (i.e. the North West and the South West). All of these limits its generalization to the entire population of Cameroonian women. The study could also be limited to the fact that it was based on self-report – women were not assessed on their ability to correctly perform BSE, which may have led to the overestimation of their knowledge on how to perform BSE.

## Conclusion

Our findings indicate that a majority of female students in the University of Buea do not practice breast self-examination as a screening method for the early detection of breast cancer. Also a majority of the students have never been to any health facility to have their breast examined. The attitude of the students was observed to be moderately in favour of BSE but the knowledge on BSE was generally unsatisfactory which could have affected the practice of BSE by these young women. Sensitization campaigns using the audiovisual media and other programs designed to create awareness about BSE should be intensified in order to change the attitude of young women in the study area towards the practice of BSE in the prevention of breast cancer.

## Additional files

Additional file 1: Table S1. Other indicators used to evaluate the knowledge of the 166 respondents. Additional file 2: Table S2. Other indicators used to evaluate the attitude of the 166 respondents toward BSE.

## Abbreviation

BSE: Breast self-examination
