# Supplementary material for: Knowledge, attitude and practice of breast self-examination among female undergraduate students in the University of Buea
Source: BMC Res Notes. 2015 Feb 15;8:43. doi: 10.1186/s13104-015-1004-4 (PMC4414436; doi:10.1186/s13104-015-1004-4)
Supplement: Additional file 1: Table S1. — Other indicators used to evaluate the knowledge of the 166 respondents. [file 13104_2015_1004_MOESM1_ESM.docx]

**Additional file 1: Table S1: Other indicators used to evaluate the knowledge of the 166 respondents**

| Knowledge | Responds | Frequency | % |
| --- | --- | --- | --- |
| Know BSE is not the only method for early detection of breast cancer | Yes | 121 | 72.9 |
|  | No | 45 | 27.1 |
| Know the age to start BSE | Yes | 82 | 49.4 |
|  | No | 84 | 50.6 |
| Know when women with regular menstruation should perform BSE | Yes | 27 | 16.3 |
|  | No | 139 | 83.7 |
| Know women should perform BSE at a specific day every month | Yes | 67 | 40.4 |
|  | No | 99 | 59.6 |
| Know BSE is performed only around the breast | Yes | 98 | 59.0 |
|  | No | 68 | 41.0 |
| Know women with menopausal problems are at high risk of breast cancer | Yes | 89 | 53.6 |
|  | No | 77 | 46.4 |
| Know women Should have BSE even if they do not feel any abnormality | Yes | 106 | 63.9 |
|  | No | 60 | 36.1 |
| Know the breast should be felt by the three fingers continuing without lifting fingers | Yes | 91 | 54.8 |
|  | No | 75 | 45.2 |
| Know that when one breast has a history of cancer, the other is also much likely to have | Yes | 116 | 69.9 |
|  | No | 50 | 30.1 |
