# Supplementary material for: Knowledge, attitude and practice of breast self-examination among female undergraduate students in the University of Buea
Source: BMC Res Notes. 2015 Feb 15;8:43. doi: 10.1186/s13104-015-1004-4 (PMC4414436; doi:10.1186/s13104-015-1004-4)
Supplement: Additional file 2: Table S2. — Other indicators used to evaluate the attitude of the 166 respondents toward BSE. [file 13104_2015_1004_MOESM2_ESM.docx]

**Additional file 2: Table S2: Other indicators used to evaluate the attitude of the 166 respondents toward BSE**

| Attitude | Response | Frequency | % |
| --- | --- | --- | --- |
| BSE is useless | Agree | 16 | 9.6 |
|  | Disagree | 129 | 77.7 |
|  | Unsure | 21 | 12.7 |
| Screening for the early stage of breast cancer is the duty of doctors and nurses | Agree | 92 | 55.5 |
|  | Disagree | 59 | 35.5 |
|  | Unsure | 15 | 9.0 |
| BSE is complicated, a waste of time and does not give accurate results | Agree | 17 | 10.2 |
|  | Disagree | 130 | 78.3 |
|  | Unsure | 19 | 11.4 |
| Having a breast removed due to cancer affects beauty, can motivate women to screen for breast cancer | Agree | 131 | 78.9 |
|  | Disagree | 18 | 10.8 |
|  | Unsure | 17 | 10.3 |
| When you know someone with breast cancer, you are more fearful and want to screen yourself | Agree | 119 | 71.7 |
|  | Disagree | 31 | 18.7 |
|  | Unsure | 16 | 9.6 |
|  |  |  |  |
